# Kirenol Attenuates Experimental Autoimmune Encephalomyelitis by Inhibiting Differentiation of Th1 and Th17 Cells and Inducing Apoptosis of Effector T Cells

**DOI:** 10.1038/srep09022

**Published:** 2015-03-12

**Authors:** Juan Xiao, Rongbing Yang, Lin Yang, Xiaohang Fan, Wenwei Liu, Wenbin Deng

**Affiliations:** 1 Medical College, Hubei University of Arts and Science, Xiangyang, Hubei, China; 2 Department of Biological Treatment, Handan Central Hospital, Handan, Hebei, China; 3 Department of Biochemistry and Molecular Medicine, School of Medicine, University of California, Davis, CA, USA

## Abstract

Experimental autoimmune encephalomyelitis (EAE), a model of multiple sclerosis (MS), is characterized by CNS demyelination mediated by autoreactive T cells. Kirenol, a biologically active substance isolated from *Herba Siegesbeckiae*, has potent anti-inflammatory activities. Here we investigated effects of kirenol on EAE. Kirenol treatment markedly delayed onset of disease and reduced clinical scores in EAE mice. Kirenol treatment reduced expression of IFN-γ and IL-17A in the serum and proportion of Th1 and Th17 cells in draining lymph nodes. Priming of lymphocytes was reduced and apoptosis of MOG-activated CD4+ T cells was increased in kirenol treated EAE mice. Kirenol treatment of healthy animals did not affect the lymphocytes in these non-immunized mice. Further *in* *vitro* studies showed that kirenol inhibited viability of MOG-specific lymphocytes and induced apoptosis of MOG-specific CD4+ T cells in a dose- and time-dependent manner. Kirenol treatment upregulated Bax,downregulated Bcl-2,and increased activation of caspase-3 and release of cytochrome *c*, indicating that a mitochondrial pathway was involved in kirenol induced apoptosis. Moreover, pretreatment with either a pan-caspase inhibitor z-VAD-fmk or a more specific caspase 3 inhibitor Ac-DEVD-CHO in lymphocytes reduced kirenol induced apoptosis. Our findings implicate kirenol as a useful agent for the treatment of MS.

Experimental autoimmune encephalomyelitis (EAE) is a commonly used animal model of multiple sclerosis (MS), the most common chronic neuroinflammation and demyelinating disease of the central nervous system (CNS) in humans^1^,^2^,^3^. During the course of EAE, anti-myelin autoreactive lymphocytes invade the CNS, leading to demyelination lesions and inflammation^4^. The ongoing paralysis and other neurological symptoms in EAE are difficult to manage and treat effectively. Both Th1 and Th17 cells are considered to be pathogenic T cell populations for the autoimmune inflammatory demyelination of CNS in EAE^5^,^6^,^7^,^8^,^9^, and the levels of Th1/Th1 cells control the development of EAE. Therefore, it is important to understand the mechanisms controlling the generation of Th1/Th17 cells in EAE. A number of mechanisms have been described to contribute to the maintenance of immune homeostasis in autoimmune CNS diseases. One such mechanism is activation induced cell death (AICD)^10^,^11^,^12^, a form of apoptosis or programmed cell death, and defect in this form of apoptosis may lead to development of autoimmune diseases including EAE. In T cell mediated CNS demyelination in EAE, AICD selectively eliminates the mature effector myelin-reactive T cells. Therefore, AICD contributes to down-regulation of inflammatory T cell activity and inhibits inflammatory responses that trigger the clinical symptoms of EAE.

Kirenol is a diterpenoid natural product compound isolated from *Herba Siegesbeckia*, where a gram of dried *Herba Siegesbeckia* contained 0.482–1.302 mg of kirenol^13^,^14^. The active fraction of *Herba Siegesbeckia* extracts reduced the inflammatory pathology in collagen induced arthritis and led to increased IL-2 but decreased IL-1 levels in the serum^15^. It has also been reported that kirenol possesses anti-inflammatory, anti-arthritic and immunoregulatory activities^13^,^16^,^17^. Kirenol upregulated nuclear Annexin-1, inhibited NF-κB activity and reduced expression of IL-1β to inhibit inflammatory response in arthritis^18^. Our previous study showed amelioration of autoimmune arthritis by kirenol, owing to modifying T cell balance and reduced secretion of pro-inflammatory cytokines^17^. Despite the well-documented anti-inflammatory properties of kirenol, whether kirenol ameliorates autoimmunity has not been investigated.

In the present study, we investigated the effects of kirenol and the mechanisms of action on the development of EAE. Our results showed that kirenol treatment significantly inhibited EAE. The reduced pathology of kirenol treated mice was associated with a decreased proportion of Th1/Th17 cells together with lower levels of IFN-γ and IL-17A in the serum and increased apoptosis of autoreactive lymphocytes in the draining lymph nodes (DLNs). Further *in vitro* studies showed that kirenol induced apoptosis of myelin reactive T cells and a mitochondrial pathway might be involved in the apoptosis induced by kirenol.

## Methods

### Chemicals and reagents

**Figure 1:**
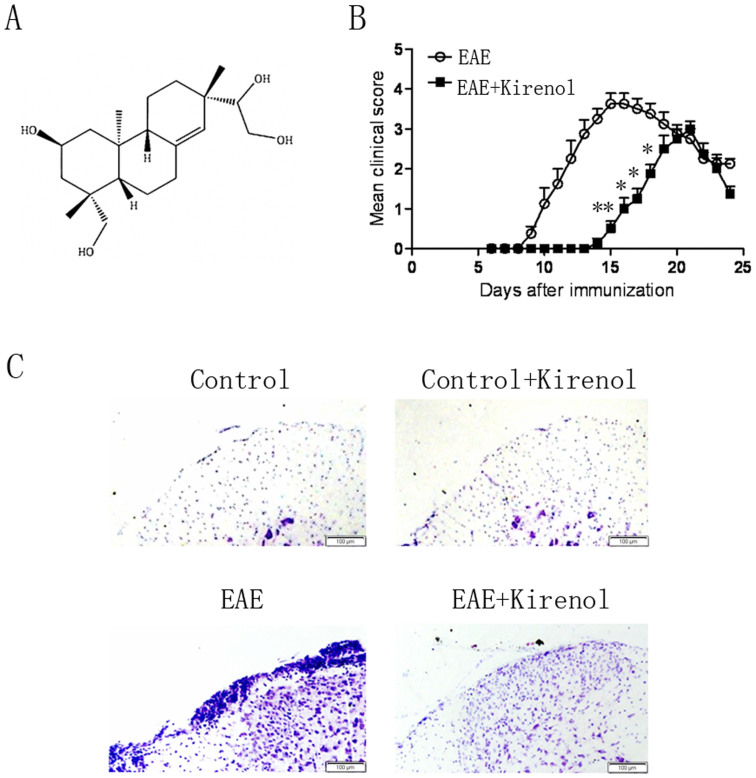
EAE disease severity is reduced in kirenol treated mice. (A) The chemical structure of kirenol. (B) EAE was induced and treated with kirenol or distilled water (n = 18 per group). Mean clinical disease scores are mean ±SD. (C) Histological sections of the lumbar spinal cord from EAE or control (non-immunized) mice at day 16 after immunization. Sections are stained with H&E and representative images are shown. The scale bar represents 100 μm.

Kirenol (purity of >99%) was obtained from the State Key Laboratory of Natural and Biomimetic Drugs at Peking University (Beijing, China). Kirenol was resuspended in distilled water and the chemical structure of kirenol shown in Fig. 1A. Antibodies detected for the following targets were purchased as indicated: Caspase-3 from Cell Signaling Technology; Bax and Bcl-2 from Santa Cruz Biotechnology; and β-actin from Sigma. Phorbol 12-myristate 13-acetate (PMA) and ionomycin were purchased from Sigma-Aldrich. Mouse Th1/Th17 phenotyping kit and Perm/Fix solution were purchased from BD Biosciences (USA). IFN-γ and IL-17A ELISA kit were purchased from eBioscience (USA). FITC-anti-CD4 and PE-anti-AV were purchased from Sungene Biotech (Tianjin, China). The MOG_35–55_ peptide (MEVGWYRSPFSRVVHLYRNGK) was synthesized by Chinese Peptide Company (Hangzhou, China). DyLight 800/DyLight 680-conjugated secondary antibodies against mouse or rabbit IgG were purchased from Rockland Immunochemicals (USA).

### Induction, clinical evaluation and treatment protocols of EAE

C57BL/6 mice were bred at the Experimental Animal Center, Peking University Health Sciences Center. All experimental procedures and protocols were approved by the Peking University Animal Ethics Committee and were performed in accordance with the institutional guidelines and regulations. EAE was induced by MOG_35–55_ in female mice used between 8 and 10 weeks of age. Briefly, each mouse was immunized subcutaneously with 300 μg of MOG_35–55_ emulsified with an equal volume of complete Freund's adjuvant (CFA, total 300 mg of *Mycobacterium tuberculosis*, strain H37RA, Difco, USA) and then injected to the caudal vein with 200 ng of pertussis toxin (PTX) (dissolved in 200 μl PBS, List Biological Laboratories, USA) at the time of immunization and 2 days later. Mice were examined for clinical scoring daily by the same investigator for 25 days after immunization in a blinded manner. Neurological assessments were reported using a five-point standardized rating scale to evaluate motor deficit: 0, no deficit; 1, tail paralysis; 2, incomplete hind limb paralysis; 3, complete hind limb paralysis; 4, complete hind limb paralysis and partial forelimb paralysis; 5, moribund state or death.

After the immunization of MOG_35–55_ on day 0, healthy (non-immunized) or EAE mice were administered daily with oral gavage of 2 mg/kg kirenol from day 0 to day 25. The no-drug control groups were administered daily with equal volumes of distilled water.

### Histological examination

Histological analysis was performed on lumbar spinal cords obtained from EAE or non-immunized mice at day 25 after immunization. Following anesthesia with intraperitoneal administration of pentobarbital, each mouse was perfused with 4% paraformaldehyde in 0.1 M phosphate buffer. Each spinal cord was carefully removed and immersed in the same fixative. The lumbar segments of each spinal cord were embedded in paraffin. Five-mm-thick sections were prepared and stained with hematoxylin-eosin (H&E).

### Flow cytometry analysis

The DLNs from the EAE or non-immunized mice were harvested at day 25, and a single cell suspension was prepared. To quantify the number of Th1/Th17 cells, cells were stimulated with PMA and ionomycin in the presence of brefeldin A for 5 h. Subsequently, cells were surface-stained with anti CD4-FITC, permeabilized with Perm/Fix solution and stained with anti-IFN-γ-PE and anti-IL-17A-PE. Isotype-matched IgG was used as a negative control. The stained cells were analyzed by FACS Caliber using Cell Quest software (BD Biosciences, USA). For detection of apoptosis, lymphocytes from DLNs with EAE or non-immunized mice were harvested and stimulated with or without of 20 μg/ml of MOG_35–55_ peptide together with different concentration of kirenol for different time. Then cells were collected and stained with anti-CD4-PE/AnnexinV-FITC, and proportion of CD4+ Annexin V+ cells were analyzed by flow cytometry.

### Lymphocyte proliferation assay and detection of cytokines in supernatant and serum

To investigate the antigen-specific lymphocytes response to MOG_35–55_, lymphocytes from DLNs at day 25 were seeded at 5 × 10^5^ cells/well in 96-well plates with RPMI 1640 containing 10% fetal calf serum (FCS) and stimulated with or without 20 μg/ml of MOG_35–55_ peptide. After 48 h, cells were pulsed with 1 μCi/well [^3^H]-thymidine (MP Biomedicals, USA) and incubated for an additional 8 h. The results are expressed as mean [^3^H] thymidine incorporation (cpm) ± standard deviation (SD).

### Cell sorting and Western blot analysis

Lymphocytes form DLNs in EAE mice were stained with anti-CD4 antibodies and sorted on a FACS instrument (BD Biosciences, USA) by gating on the CD4+ population. For western blot analysis experiment, the sorted CD4+ T cells were lysed in lysis buffer (300 mM NaCl, 50 mM Tris pH 8.0, 0.4% NP-40, 10 mM MgCl_2_, and 2.5 mM CaCl_2_) supplemented with protease inhibitors (Complete mini EDTA-free; Roche Diagnostics, Mannheim, Germany). After centrifugation, the supernatant was measured using the BCA protein assay reagent (Pierce, Rockford, IL). Then, 1 μg of total cell extracts protein was loaded onto 12.5% SDS-PAGE, transferred to nitrocellulose membrane (Amersham Pharmacia Biotech, Little Chalfont, UK), blocked by incubation with 5% non-fat milk in TBS-T buffer (10 mM Tris–HCl, pH 7.4, 150 mM NaCl and 0.1% Tween-20) for 1 h, and blotted against the different proteins using specific antibodies: anti-caspase-3, anti-Bax, anti-Bcl-2, anti-β-Actin and anti-cytochrome *c*. After washings with TBS, the protein bands were visualized using DyLight 800/DyLight 680-conjugated secondary antibodies, and the infrared fluorescence image was obtained using an Odyssey infrared imaging system (LI-COR Biosciences, USA).

### Caspase 3 activity assay

Briefly, lymphocytes from DLNs of EAE mice were incubated in a 48-well plate with 2 × 10^6^ cells per well and treated with the indicated concentration of drugs. Cells were then collected and sorted for CD4+ T cells. Caspase3 activity was measured using a caspase 3 activity assay kit (Beytime, China) according to the manufacturer's instructions. The absorbance was measured at 405 nm using a microplate reader. The experiments were carried out in triplicate.

## Results

### Kirenol suppresses the pathogenesis of EAE

After immunization of MOG_35–55_ on day 0, the mice were treated with a daily oral gavage of kirenol or saline for 25 d, and regularly graded for signs of EAE. Both the day of clinical onset and the day of peak clinical disease were delayed in kirenol treated mice compared to saline treated EAE mice. The peak clinical score of kirenol treated EAE mice was significantly lower compared with that of saline treated mice (Fig. 1A, 1B). Histological examinations of the lumbar spinal cord tissue collected on day 25 post immunization revealed that massive inflammatory infiltration was evident in the spinal cord of EAE mice. However, minimal infiltration of inflammatory lymphocytes was found in kirenol treated EAE mice (Fig. 1C). Kirenol did not affect lymphocyte infiltration in non-immunized mice (Fig. 1C). Together, these results indicated that kirenol treatment significantly reduced the demyelination of CNS during EAE.

### Kirenol decreases the differentiation of IFN-γ and IL-17A producing CD4+ T cells *in vivo*

**Figure 2:**
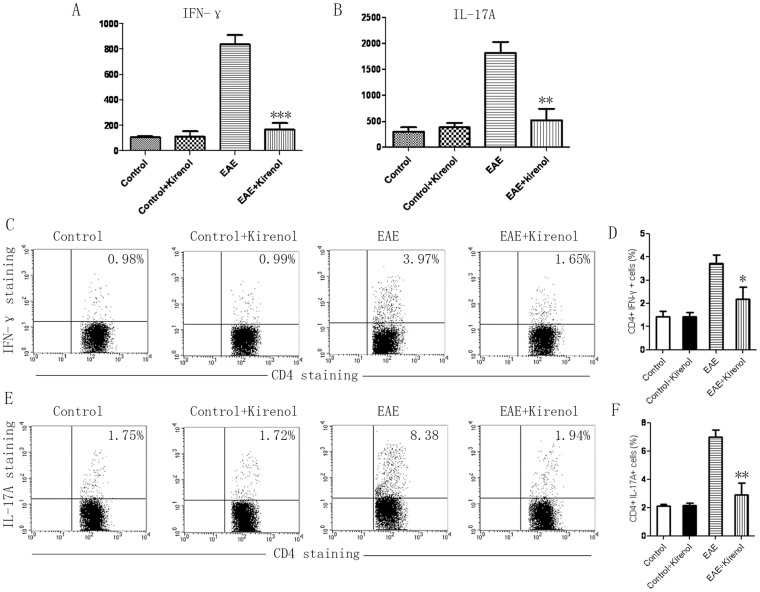
Th1/Th17 cells are reduced in kirenol treated EAE mice. Serum from EAE or control (non-immunized) mice at day 25 was obtained and the concentration of INF-γ (A) and IL-17A (B) was measured by ELISA. (C) Representative image of CD4+IFN-γ+ flow cytometry. (D) Quantification of the number of CD4+IFN-γ+ Th1 cells. (E) Representative image of CD4+IL-17A+ cells by flow cytometry. (F) Quantification of the number of CD4+IL-17A+ Th17 cells.

To investigate the immunological mechanisms associated with the reduced severity of EAE in kirenol treated mice. Serum samples were collected at 25 days after immunization and cytokine levels were measured by ELISA. Kirenol treated EAE mice produced significantly low level of serum IFN-γ and IL-17A compared with saline treated mice (Fig. 2A, 2B). The results indicated that kirenol downregulated the inflammatory Th1/Th17 response *in vivo*. Next, we examined the cellular phenotypes of EAE mice treated with kirenol and saline. Single cell suspensions were prepared from the DLNs of EAE mice at day 25 after immunization as described above, and stained for cell surface and intracellular markers by flow cytometry. Consistent with the cytokines profile observed above, there was a significant reduction in the frequency of IFN-γ+ and IL-17A+ cells in the DLNs from EAE mice treated with kirenol (Fig. 2C, 2D, 2E, 2F) compared to mice treated with saline. Kirenol did not affect concentration of cytokines in healthy (non-immunized) animals. Together, these data indicated *in vivo* downregulation of inflammatory Th1/Th17 cells in the EAE mice treated with kirenol.

### Kirenol reduces effector and effector memory CD4+ T cells in the DLNs *in vivo*

**Figure 3:**
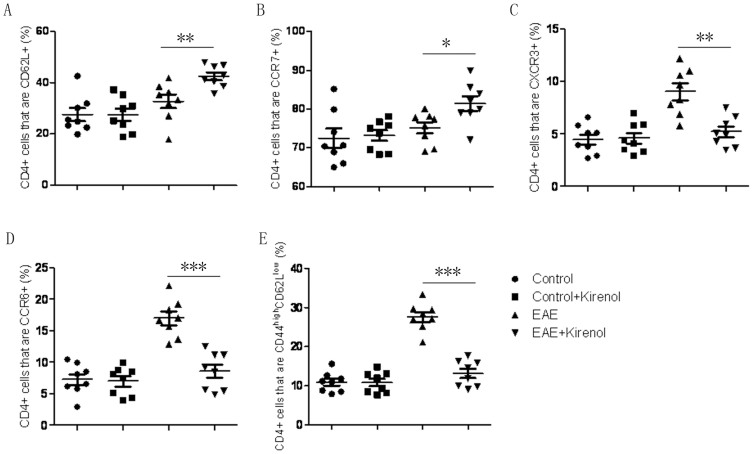
T cell priming is reduced in kirenol treated EAE mice. Lymphocytes were obtained from DLNs of EAE or control (non-immunized) mice at day 25 after immunization. Priming of T cells was reduced in EAE mice treated with kirenol as demonstrated by higher proportion of CD62L+ (A) and CCR7+ (B) T cells that gated on CD4+ T cells and lower proportion of CXCR3+ (C) or CCR6+ (D) T cells that gated on CD4+ T cells. (F) The proportion of memory T cells (CD4^+^CD44^high^CD62L^low^) in DLNs was lower in kirenol treated EAE mice.

In secondary lymphoid organs such as DLNs, priming of CD4+ T cells plays an important role in the development of autoreactive T cell responses and subsequent inflammatory pathology in EAE. To determine if the diminished clinical presentation of autoimmunity in kirenol treated mice was related to defects in T cells priming, lymphocytes obtained from DLNs were gated on CD4+ T cells, and CD62L, CCR7, CXCR3, CCR6 and CD44^high^/CD62L^low^ expression on CD4+ T cells were analyzed by flow cytometry. As shown in Fig. 3A and Fig. 3B, a higher proportion of CD4+ T cells in DLNs of kirenol treated mice expressed markers of naïve T cells (CD62L and CCR7). Furthermore, a lower proportion of CD4+ T cells were positive for markers of effector T cells such as the chemokine receptors CXCR3 and CCR6 (Fig. 3C, 3D), and expressed an effector/memory phenotype (CD44^high^/CD62L^low^) (Fig. 3E). Kirenol did not affect CD4+ T cells from non-MOG immunized mice. These results indicated less efficient T cell (MOG-specific) activation in kirenol treated mice.

### Kirenol induces apoptosis of MOG-specific T cells *in vivo* and *in vitro*

**Figure 4:**
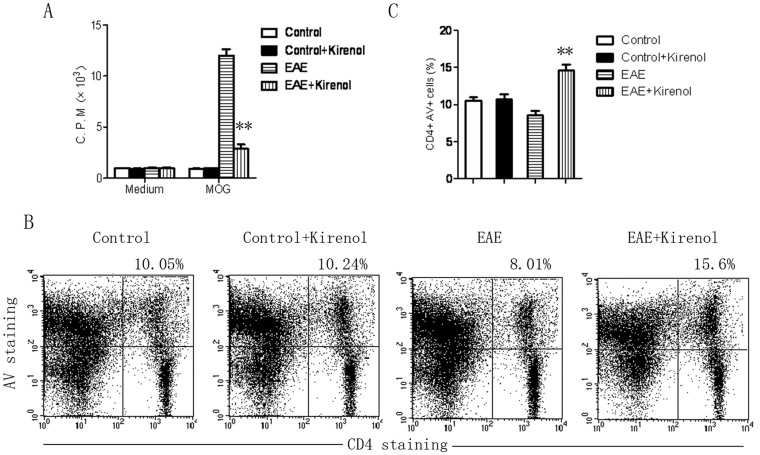
T cell apoptosis is reduced in EAE mice treated with kirenol. Lymphocytes of DLNs were obtained from EAE or control (non-immunized) mice at day 25. Lymphocytes were stimulated with or without MOG_35–55_ for 48 h, and then cell proliferation was measured by [^3^H]-thymidine incorporation assay. (B) Representative images of CD4+AV+ flow cytometry in DLNs are shown. (C) Quantification of the number of CD4+AV+ cells in DLNs.

Next, the response of autoreactive lymphocytes to MOG_35–55_ in DLNs from EAE mice treated with saline or kirenol was measured by [^3^H]-thymidine incorporation assay. Significantly lower [^3^H]-thymidine incorporation was seen in cells from mice treated with kirenol (Fig. 4A), indicating impaired immune priming of effector T cells in kirenol treated mice. The defect in T cell priming could be a result of impaired T cell proliferation or survival, or a combination of both. We then assessed the apoptosis of CD4+ T cells from DLNs of EAE mice treated with kirenol. We observed that a higher proportion of apoptotic cells were present in DLNs of kirenol treated mice compared to saline treated mice (Fig. 4B), indicating that kirenol promoted apoptosis of CD4+ T cells.

**Figure 5:**
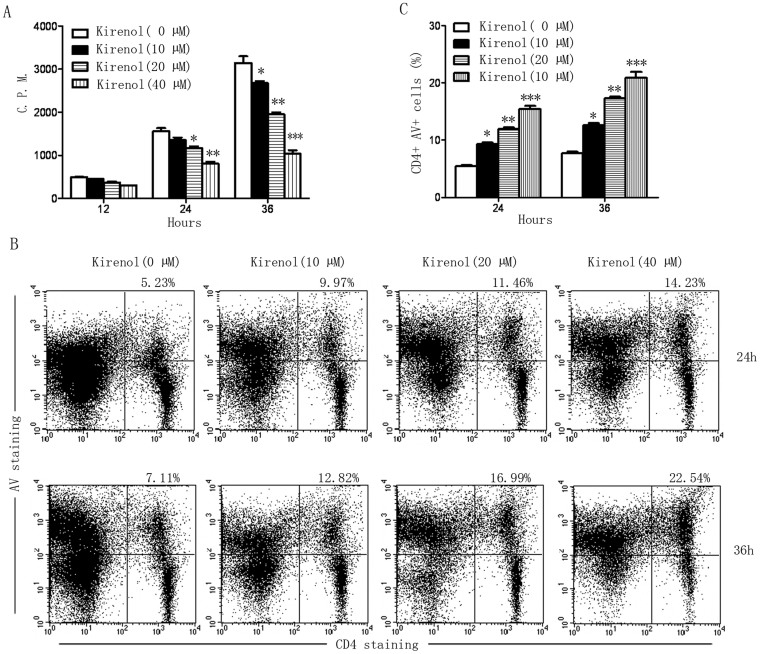
Kirenol induces T cell apoptosis *in vitro*. Lymphocytes of DLNs were obtained from EAE mice at day 25. Lymphocytes were stimulated with MOG_35–55_ together with increasing concentrations of kirenol for 12 h, 24 h and 36 h, and then cell proliferation was measured by [^3^H]-thymidine incorporation assay. (B) Representative images of CD4+AV+ flow cytometry in DLNs that were stimulated with MOG_35–55_ together with increasing concentrations of kirenol for 24 h and 36 h are shown. (C) Quantification of the number of CD4+AV+ cells.

We further investigated the effects of kirenol in antigen-specific lymphocytes proliferation *in vitro*. Lymphocytes of DLNs from EAE mice were incubated with MOG_35–55_ together with increasing concentrations of kirenol. As shown in Fig. 5A, lymphocytes proliferation was reduced in a time-dependent manner when cultured in the presence of kirenol, indicating that kirenol inhibited priming of effector cells. To assess if the observed reduction in cell viability was due to apoptosis, lymphocytes were incubated with MOG_35–55_ together with increasing concentrations of kirenol, and the percentage of CD4+ Annexin V+ was analyzed by flow cytometry. As shown in Fig. 5B, the proportion of CD4+ T cells cultured with kirenol underwent apoptosis in a concentration-dependent manner, indicating that kirenol promoted apoptosis of CD4+ T cells.

### Kirenol induces MOG-specific T cells apoptosis via a mitochondrial pathway

**Figure 6:**
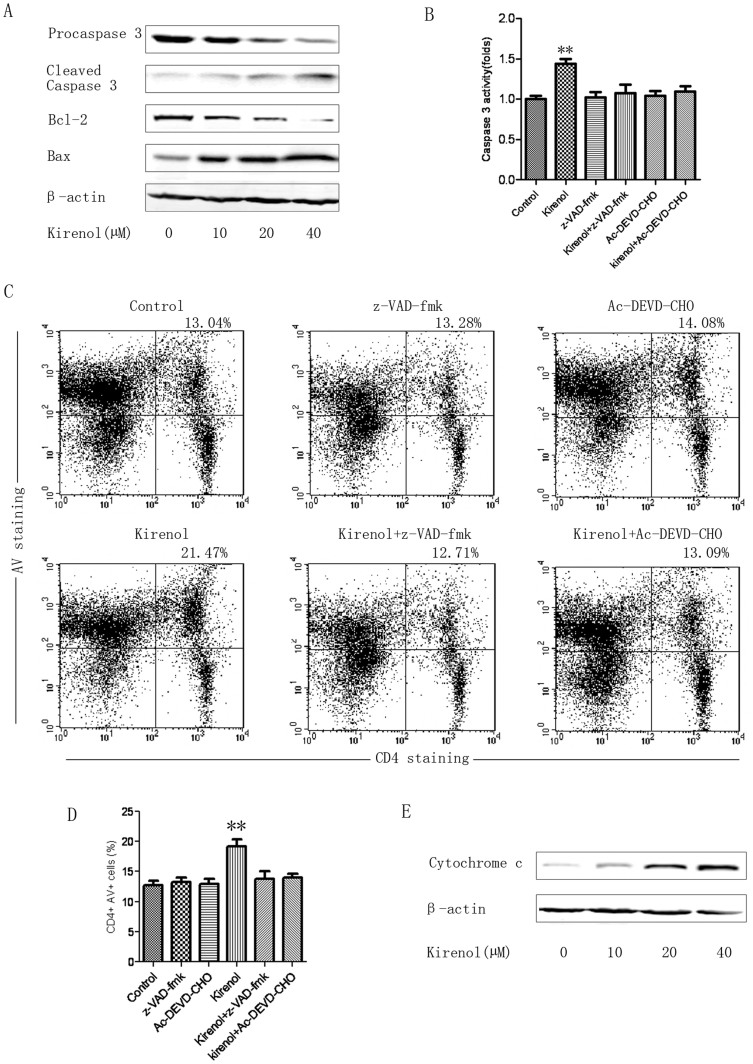
A mitochondria-dependent pathway is involved in the apoptosis induced by kirenol. Lymphocytes from DLNs in EAE mice at day 25 were stimulated with MOG_35–55_ together with different concentrations of kirenol for 36 h and then gated on the CD4+ T cells. Next, the expression of caspase 3, Bax, Bcl-2 (A) and cytochrome *c* (E) in CD4+ T cells were analyzed by western blot. Gels have been run under the same experimental conditions. (B) Lymphocytes were pretreated with or without either the pan-caspase inhibitor z-VAD-fmk (10 μM) or a more specific caspase 3 inhibitor Ac-DEVD-CHO (10 μM) for 4 h followed by treatment with kirenol (20 μM) for 36 h and the gated on CD4+ T cells, and the relative activity of caspase 3 was measured using a caspase 3 activity assay. The representative images of CD4+AV+ flow cytometry (C) and quantification of the number of CD4+AV+ cells (D) are shown.

To further investigate the mechanisms of the induction of apoptosis in MOG-specific T cells induced by kirenol, lymphocytes from DLNs in EAE mice were stimulated with MOG_35–55_ together with the indicated concentrations of kirenol for 36 h and then gated on CD4+ T cells and measured for the expression of apoptosis-related proteins by western blot analysis. The results showed that Bax expression was dose-dependently increased, whereas Bcl-2 expression was dose-dependently decreased. Furthermore, a dose-dependent elevation of cleaved caspase 3 was seen in kirenol treated CD4+ T cells (Fig. 6A). Next, lymphocytes were stimulated with MOG_35–55_ together with either the pan-caspase inhibitor z-VAD-fmk (10 μM) or a more specific caspase 3 inhibitor Ac-DEVD-CHO (10 μM) for 4 h and with kirenol (20 μM) for another 36 h and then gated on CD4+ T cells. We found that the relative activities of caspase 3 were increased in CD4+ T cells treated with kirenol only (Fig. 6B). Furthermore, CD4+ T cells pretreated with z-VAD-fmk or Ac-DEVD-CHO strongly reduced kirenol-induced apoptosis (Fig. 6C, D). The expression of cytochrome *c* released from mitochondria into the cytoplasm during apoptosis was also examined. As shown in Fig. 6E, kirenol increased the expression of cytochrome *c* in a dose dependent manner. These findings indicated that a mitochondria-dependent pathway was involved in apoptosis induced by kirenol.

## Discussion

This study revealed novel anti-inflammatory therapeutic effects of kirenol in a CNS autoimmune disease. First, we showed that it delayed the onset and decreased the clinical scores of EAE in mice. Second, we demonstrated that the amelioration in EAE pathology was due to decrease in the proportion of Th1/Th17 cells, but also attributed to T cell activation and higher levels of AICD in T cells. Third, we found that a mitochondrial pathway was involved in apoptosis induced by kirenol.

EAE is mediated by the auto-myelin reactive T lymphocytes that enter into the CNS and secrete pro-inflammatory cytokines, leading to the formation of inflammatory and demyelinating lesions^19^. Th1 cells were considered to be the critical T-helper cells for the autoimmune inflammation in the brain, and then the discovery of Th17 cells critically improved our understanding of the cellular basis of EAE pathogenesis^8^,^20^. Increasing evidence has shown that both Th1 and Th17 cells drive the pathogenesis of EAE, but neither cell type can induce the same extent of EAE without contribution from the other^21^,^22^,^23^. Regulatory T (Treg) cells are known as a subset of CD4+ T cells that are essential for controlling autoimmune inflammation by suppressing autoreactive T cells, and are able to inhibit the pathogenesis of EAE^24^,^25^. In the present study, the reduced clinical signs and pathologies in EAE mice treated with kirenol were unlikely due to the enhanced Treg response, as there was no apparent increase in the proportion of Treg cells during the EAE course,. We showed that the generation of Th1/Th17 cells and secretion of IFN-γ and IL-17A were reduced in kirenol treated mice.

During autoimmune response, the magnitude of autoreactive T cell response against a specific antigen is determined by the balance between the increasing number of antigen-specific T cells and loss of antigen-specific T cells by AICD. A previous report showed that administration of apoptosis inhibitors resulted in impaired recovery and earlier relapse in EAE by suppressing apoptosis of inflammatory cells^26^. However, other studies reported that induction of autoreactive T cell apoptosis reduced disease severity in EAE^27^,^28^. Our study showed that kirenol inhibited the survival of mature antigen-specific T cells, as a higher proportion of naïve T cells but a lower proportion of effector/memory T cells were observed in the DLNs of kirenol treated mice. Further *in vivo* and *in vitro* studies demonstrated that kirenol inhibited the survival of antigen-specific T cells by inducing apoptosis.

In lymphocytes, apoptosis can be triggered by the extrinsic pathway activated by the death receptor and the intrinsic pathway activated by Blc-2 family members and caspase cascades within the mitochondria^29^. In the intrinsic pathway of apoptosis, activation of Bax leads to mitochondrial dysfunction and cell death. The Bax protein can form heterodimers with the apoptosis inhibitor Bcl-2, and the relative levels of these two molecules determine the death of the cells^30^. In the present study, kirenol treatment increased the expression of Bax but decreased the expression of Bcl-2 in lymphocytes of DLNs in a dose-dependent manner. An increased ratio of pro- and anti-apoptotic protein expression, such as Bax/Bcl-2 ratio, may lead to the loss of mitochondrial membrane potential. Cytochrome *c* is then released from dysfunctional mitochondria and accumulates in the cytosol to form a complex with Apaf-1. Caspase 3 is known to be cleaved in the intrinsic apoptotic pathway^31^. Our study showed that caspase 3 was activated by kirenol in a dose-dependent manner. The caspase 3 inhibitor Ac-DEVD-CHO reduced apoptosis in antigen-specific T cells induced by kirenol, indicating that the mitochondrial pathway plays an important role in the apoptosis of autoreactive T cells induced by kirenol.

In conclusion, the present study demonstrated the therapeutic potential of kirenol in MS and other autoimmune CNS diseases. Kirenol reduced the severity of EAE by inhibiting differentiation of Th1/Th17 cells and inducing apoptosis of MOG-specific T cells through a mitochondria-dependent pathway. This novel mechanism of action may account for the therapeutic potential of kirenol in MS by elimination of cells responsible for CNS inflammation.

## Author Contributions

J.X., R.Y., X.Y., L.Y. and X.F. carried out the experiments. W.L. provided critical input and co-supervised the study. J.X. and W.D. designed experiments and wrote the paper.
